# Supplementary material for: Environmental disclosure practices in mixed ownership models: A study of Chinese private enterprises
Source: PLoS One. 2023 Nov 30;18(11):e0295399. doi: 10.1371/journal.pone.0295399 (PMC10688842; doi:10.1371/journal.pone.0295399)
Supplement: Attachment — Submitted filename: Review Result_PONE-D-23-25299.docx [file pone.0295399.s001.docx]

**REVIEW RESULT**

**Manuscript_PONE-D-23-25299t: Environmental Disclosure Practices in Mixed Ownership Models: A Study of Chinese Private Enterprises**

Reviewer: Dr. Le Quynh Lien

1. **OBJECTIVE:** The objective of the article is met with the paper’s scope.
2. **INTRODUTION:** It is important to provide clarity regarding your research questions, objectives, the motivation behind your study, both in terms of background and theory, as well as the empirical motivation. Additionally, you should outline how your study contributes to the existing literature. This can be achieved by explicitly stating your research questions and objectives, identifying potential theoretical and background motivations or gaps, and elucidating how your research adds to the body of knowledge in this field. Authors need to elaborate on the novel contribution of this study compared to previous research, shedding light on why the topic of "Environmental Disclosure Practices in Mixed Ownership Models" was chosen, and how the choice of China as the context differs from other studies addressing the same topic within a similar context. Please see the following papers:
   1. Kilincarslan, E., Elmagrhi, M.H. and Li, Z. (2020), "Impact of governance structures on environmental disclosures in the Middle East and Africa", Corporate Governance, Vol. 20 No. 4, pp. 739-763. <https://doi.org/10.1108/CG-08-2019-0250>
   2. Bin, L., Lei, X., Ron, P. M., Xin, L., and Ailing, Pan. (2022), “Mixed-Ownership Reform and Private Firms’ Corporate Social Responsibility Practices: Evidence From China, Business & Society, Vol. 61(2) 389–418. <http://doi.org/10.1177/0007650320958762>
   3. Meng, X.H., Zeng, S.X., Jonathan, J. S., Qi, G.Y. and Zhang. Z.B. (2014), "The relationship between corporate environmental performance and environmental disclosure: An empirical study in China", Journal of Environmental Management, Vol. 145, p.357-367, <https://doi.org/10.1016/j.jenvman.2014.07.009>.

**3.	LITERATURE REVIEW: PRIOR STUDIES AND HYPOTHESES DEVELOPMENT**

-	COMPREHENSIVE PRIOR LITERATURE: There are appropriate and adequate references to related and previous research. Literature review and hypothesis development should be discussed in more detail, in my opinion. Please offer a comprehensive theoretical framework that will explain the underlying assumptions and hypotheses of interest. Please use both seminal (old) and recently (newly) published studies to support your argument, making sure to clearly state how they contribute to the connection between the dependent and independent variables.

Review of the literature and development of hypotheses: Please strengthen your hypotheses drawing from theory, (ii) referencing empirical research, (iii) considering the study setting and context, and (iv) finally putting them into place. You should do this by referencing both classic (previously published) and contemporary (recently published) studies. Most of the recent related studies mentioned in your study are just in 2020 and earlier. It should be updated.

-	HYPOTHESIS DEVELOPMENT: The hypotheses have been developed based on previous studies.

**4.	METHODOLOGY, RESEARCH DESIGN AND METHODS**

-	The research methodology in this study is sound.

-	Please explain why the authors chose to calculate the maximum score of 37 points for companies to determine their EID and then divide the EID scores of sample firms by this maximum score to obtain the company's EID index. The authors should reference reputable studies that have employed this method to measure EID scores.

-	Further evidence is needed to support the argument that there is a lag in the effects of participation by state-owned capital on private firms' EID, suggesting that there is no immediate effect in the year of participation. This would enhance the persuasiveness of using lag variables when conducting Robustness tests.

**5. FINDINGS & DISCUSSION**

-	In the research section, it is important for the authors to provide clear conclusions regarding the hypotheses and indicate which hypotheses have been accepted or rejected. Furthermore, the authors should discuss how their results align with prior studies or use relevant theories to explain the observed outcomes.

-	Below each result table, there should be additional Notes regarding the applied regression method, dependent variables, independent variables, and the significance symbols *, **, and *** in the result tables.

- Table 3 requires a more detailed description. The authors should provide a more in-depth analysis of the significance of the minimum, maximum, mean, and standard deviation values of the dependent and independent variables in the model.
- Specify the Multivariate regression methods more explicitly in Table 5.

**6. ENGLISH LANGUAGE AND STYLE:**

It has some minor mistakes. The article could be proofread by a native speaker to enhance the quality of communication.

**7. OTHER SUGGESTIONS FOR THE AUTHOR(S):**

-	Keywords should be arranged in alphabetical order.

-	The list of references should adhere to the required citation style. For example:

1. Syed AM, Ntim CG. Environment, social, and governance (ESG) criteria and preference of managers. Cogent Bus Manage. 2017; 4:1340820. <https://doi.org/10.1080/23311975.2017.1340820>.

2. Boonlert-U-Thai K, Meek JK, Nabar S. Earnings attributes and investor-protection: international evidence. Int J Account. 2006; 41:327–57. https://doi.org/10.1016/J.INTACC.2006.09.008.
