# Supplementary material for: Environmental disclosure practices in mixed ownership models: A study of Chinese private enterprises
Source: PLoS One. 2023 Nov 30;18(11):e0295399. doi: 10.1371/journal.pone.0295399 (PMC10688842; doi:10.1371/journal.pone.0295399)
Supplement: Attachment — Submitted filename: Response to Reviewers.docx [file pone.0295399.s002.docx]

**Response to Reviewers**

Dear editor and reviewer:

Hello everyone, thank you very much to the editors and reviewers for carefully reviewing this article and providing valuable suggestions for revisions. I have made careful revisions and supplements according to the reviewer's comments, and all modifications have been highlighted in red font in the revised manuscript.

I have made revisions and supplements to the attached modification comments item by item, as follows:

**Reviewer#1, Concern # 1:** The objective of the article is met with the paper’s scope.

**Author response:** Thank you for the reviewer's approval

**Reviewer#1, Concern # 2:INTRODUTION:** It is important to provide clarity regarding your research questions, objectives, the motivation behind your study, both in terms of background and theory, as well as the empirical motivation. Additionally, you should outline how your study contributes to the existing literature. This can be achieved by explicitly stating your research questions and objectives, identifying potential theoretical and background motivations or gaps, and elucidating how your research adds to the body of knowledge in this field. Authors need to elaborate on the novel contribution of this study compared to previous research, shedding light on why the topic of "Environmental Disclosure Practices in Mixed Ownership Models" was chosen, and how the choice of China as the context differs from other studies addressing the same topic within a similar context. Please see the following papers:

**Author response:** Based on the comments of the reviewers, I have reorganized the introduction of the article. Based on the research background, the research questions and objectives of this study are proposed, as well as why the environmental disclosure practice under the mixed ownership model is chosen. At the same time, modifications were made to the research contributions, detailing the differences between this study and other studies.

**Author action:** The following is a modified portion of the paper in response to the comments：

These measures underscore China’s growing commitment to EID, positively influencing the EID practices of listed firms. However, compared to developed countries, EID in China remains in its nascent stage, primarily due to the lack of robust laws, regulations, and punishment mechanisms for corporate EID. This deficiency is particularly pronounced among private enterprises [4]. Zeng et al. [5] investigated the EID status of China's listed manufacturing enterprises and discovered that the EID was influenced by the nature of ownership of the majority shareholder, industry, and company size, with commercial companies having lower EID rates than state-owned counterparts. In the Chinese context, EID of listed companies is mostly not mandatory and reflecting their sense of social responsibility. Compared to state-owned enterprises, private enterprises do not have excessive policy burdens. Some private enterprises often disclose environmental information based on strategic needs [6], with varying degree of awareness regarding self-disclosure of environmental information. In addition, environmental governance is marked by high investments, high risks, and long timeframes, making it difficult to achieve results in a short period of time [7]. Private enterprises often face resource acquisition limitations [8], resulting in insufficient investments in environmental governance making it difficult to improve the level and quality of EID. Private businesses are a key component of China's economic and social development and are essential for advancing high-quality sustainable economic growth and environmental protection. Thus, exploring the determinants of EID by private enterprises within the Chinese context has become a major issue that necessitates collaboration among the government, market, and corporate entities.

Recently, China has been actively implementing changes to facilitate mixed ownership. Microeconomic mixed ownership involves intertwining state-owned, collective, and non-public capital through mutual integration and cross-shareholding. Hence, private funds may invest in state-owned enterprises, or state-owned capital may invest in private companies (including private firms) [9]. State-owned capital participation in private enterprises carries a two-fold impact. On one hand, the equity affiliation of private firms with state-owned shareholders strengthen ties with the government and may facilitate access to resources and reputational capital, thereby enhancing potential capital investment in environmental governance. On the other hand, state-owned shareholders, driven by political needs and social norms, may exert pressure on private firms for environmental governance. Concurrently, they convey a sense of environmental awareness, thereby increasing the likelihood that private firms will partake in EID. Therefore, it is urgent for us to further confirm whether mixed ownership reform can promote private enterprises to actively disclose environmental information.

Using a sample of privately listed Chinese companies from 2010 to 2020, this study empirically examines the effect of state ownership of capital on the EID. This research contributes to the field in two significant ways. Firstly, while previous literature has explored the internal factors that affect corporate EID in China, mainly focusing on company characteristics [10], executive characteristics [11], shareholder pressure [12] etc. Few scholars have studied the impact of equity structure on corporate EID. Moreover, previous research on the equity structure of enterprises has predominantly focused on a single dimension of state-owned and non-state-owned equity [13, 14]. This article examines the level of EID in private enterprises under mixed equity in the Chinese context, demonstrating that mixed equity can effectively integrate resources and improve governance mechanisms, thereby altering the EID decisions of enterprises, and to some extent, enriching literature research on enterprise environmental information disclosure. Secondly, existing literature on mixed ownership reform mainly focuses on state-owned enterprises and explores the consequences of private capital entering state-owned enterprises, such as corporate performance [15], corporate innovation[8], excessive perquisite consumption by executives [16], green transformation [17], with relatively limited attention given to mixed reforms involving private enterprises. A small number of scholars have explored the impact of private enterprises reform on corporate default risk [18] and social responsibility [19], etc. Surprisingly, the mechanism by which the disclosure of environmental information in private enterprises is influenced has remained unexplored. This paper takes private enterprises as the participating subject, analyzes the mechanism by which state-owned capital participation affects the EID of private firms from the perspectives of resource effects and governance effects, thus supplementing research on the path of state-owned capital participation to promote the EID of private firms.

**Reviewer#1, Concern # 3: LITERATURE REVIEW: PRIOR STUDIES AND HYPOTHESES DEVELOPMENT**

-	COMPREHENSIVE PRIOR LITERATURE: There are appropriate and adequate references to related and previous research. Literature review and hypothesis development should be discussed in more detail, in my opinion. Please offer a comprehensive theoretical framework that will explain the underlying assumptions and hypotheses of interest. Please use both seminal (old) and recently (newly) published studies to support your argument, making sure to clearly state how they contribute to the connection between the dependent and independent variables.

Review of the literature and development of hypotheses: Please strengthen your hypotheses drawing from theory, (ii) referencing empirical research, (iii) considering the study setting and context, and (iv) finally putting them into place. You should do this by referencing both classic (previously published) and contemporary (recently published) studies. Most of the recent related studies mentioned in your study are just in 2020 and earlier. It should be updated.

-	HYPOTHESIS DEVELOPMENT: The hypotheses have been developed based on previous studies.

**Author response:** Based on the comments of the reviewers, I have made revisions to the literature review and hypothesis development of the article. Firstly, a literature review on environmental information disclosure was supplemented from three aspects, including environmental performance and environmental information disclosure, the influencing factors of environmental information, and the economic results of environmental information disclosure. Secondly, the theoretical framework was elaborated. Finally, the hypothesis development was enriched, and relevant references were updated to include research after 2020.

**Author action:** The following is a modified portion of the paper in response to the comments：

Current research in the field of EID has primarily focused on three key areas. Firstly, analyzing the correlation between environmental performance and environmental information disclosure has not yet reached a consistent conclusion. Acar and Temiz [20] supported a positive correlation between environmental performance and EID, and they argued that better environmental performers are more willing to participate in EID. Patten's study suggested a negative relationship between environmental performance and EID, arguing that firms use environmental information as a legitimate tool, and that companies with poor environmental performance will disclose more positive environmental information [21]. Meng et al. [22] found a non-linear relationship between corporate environmental performance and EID through content analysis of 533 listed companies. Secondly, existing literature has explored the economic consequences of EID, including financial performance [23], green innovation [24], and stock price collapse risk [25]. The third aspect of research has been focused on the factors influencing EID. It mainly focuses on both external and internal aspects of enterprises. From an external perspective of enterprises, formal environmental regulatory policies force enterprises to comply with regulations in order to obtain legitimacy, resulting in a tendency to disclose high-quality environmental information [26]. Under the informal regulatory pressure of media supervision [27] and stakeholders [28], companies usually disclose environmental information in order to maintain their reputation. In addition, the degree of EID by enterprises may also vary due to policy uncertainty [29] and the level of industry and market competition [28]. From an internal perspective, factors such as firm characteristics [10], profitability [30], board characteristics [31, 32], and corporate governance [33] can all affect corporate EID to some extent.

These are newly added literature reviews, assuming that the development part is reflected in the article, they will not be listed one by one.

**Reviewer#1, Concern # 4: METHODOLOGY, RESEARCH DESIGN AND METHODS**

-	The research methodology in this study is sound.

-	Please explain why the authors chose to calculate the maximum score of 37 points for companies to determine their EID and then divide the EID scores of sample firms by this maximum score to obtain the company's EID index. The authors should reference reputable studies that have employed this method to measure EID scores.

-	Further evidence is needed to support the argument that there is a lag in the effects of participation by state-owned capital on private firms' EID, suggesting that there is no immediate effect in the year of participation. This would enhance the persuasiveness of using lag variables when conducting Robustness tests.

**Author response:** Based on the research of scholars such as Chen, an evaluation index for environmental information disclosure was constructed. According to the content analysis method, the optimal score for environmental information disclosure was 29. The rules are as follows: A score of zero means there was no information disclosed, while a score of 1 signifies the presence of information. For environmental liabilities and environmental governance and performance, zero means no description, 1 represents a general qualitative description, and a score of 2 indicates a quantitative description.

In the robustness test section, we have replaced the lagged test method with the instrumental variable method. We believe that the instrumental variable method is more persuasive in alleviating endogeneity problems caused by omitted variable bias and bidirectional causality.

**Author action:** We have provided a detailed explanation of the optimal score for environmental information disclosure. We referred to the study by Chen et al. [10] using the "content analysis method" to quantify the EID of enterprises. We divided the environmental information disclosed by the company into six parts. we calculated the maximum score of 29 points for companies with respect to EID (The specific assignment of indicators is shown in Table 1) and then divided sample firms' EID scores by the maximum score of EID to obtain the company's EID index. The calculation formula is EID = 100* ( EIDI/29).

The average value of state-owned equity share of private enterprises in the same industry in the same year was used as an instrumental variable and regressed by two-stage least squares estimation (2SLS). The regression results are shown in Table 6, confirming the hypotheses.

Table 1. Corporate EID indicators.

| Level 1 Indicators | Secondary indicators | Numerical value | | |
| --- | --- | --- | --- | --- |
|  |  | Undisclosed | Qualitative Disclosure | Quantitative Disclosure |
| Environmental Liabilities | Discharge of wastewater | 0 | 1 | 2 |
|  | Emissions of SO_2_ | 0 | 1 | 2 |
|  | Emissions of CO_2_ | 0 | 1 | 2 |
|  | Emissions of smoke and dust | 0 | 1 | 2 |
|  | Generation of solid waste in industry | 0 | 1 | 2 |
| Environmental Governance and Performance | Techniques for lowering emissions of exhaust gas | 0 | 1 | 2 |
|  | Wastewater abatement treatment | 0 | 1 | 2 |
|  | Cleaner production implementation | 0 | 1 | 2 |
|  | Use and disposal of solid waste. | 0 | 1 | 2 |
| Disclosure Vehicle | List Companies' Annual Reports | Have for 1, not have for 0 | | |
|  | Social Responsibility Report |  |  |  |
| Environmental Costs and Inputs | Environmental expenses |  |  |  |
|  | Total environmental investment |  |  |  |
| Environmental Management | Training and education in environmental studies |  |  |  |
|  | The environmental incident response mechanism |  |  |  |
|  | Environmental concept |  |  |  |
|  | Environmental goals |  |  |  |
| Government Regulation and Institutional Certification | The implementation of the " triple concurrent" system |  |  |  |
|  | ISO environmental certification implementation |  |  |  |
|  | Environmental honors |  |  |  |

Table 6. Instrumental variable results.

| Variable | First-stage | | Second-stage | |
| --- | --- | --- | --- | --- |
|  | (1) | （2） | |  |
|  | State | EID | |  |
| IVState | 0.810*** |  | |  |
|  | (7.11) |  | |  |
| State |  | 1.031*** | |  |
|  |  | (4.99) | |  |
| Constant | -0.101*** | -0.708*** | |  |
|  | (-8.83) | (-18.53) | |  |
| Controls | Yes | Yes | |  |
| Year | Yes | Yes | |  |
| Industry | Yes | Yes | |  |
| N | 14339 | 14339 | |  |
| adj. R^2^ | 0.079 | 0.089 | |  |

Note: *** p<0.01, ** p<0.05, * p<0.1.

**Reviewer#1, Concern # 5: FINDINGS & DISCUSSION**

-	In the research section, it is important for the authors to provide clear conclusions regarding the hypotheses and indicate which hypotheses have been accepted or rejected. Furthermore, the authors should discuss how their results align with prior studies or use relevant theories to explain the observed outcomes.

-	Below each result table, there should be additional Notes regarding the applied regression method, dependent variables, independent variables, and the significance symbols *, **, and *** in the result tables.

- Table 3 requires a more detailed description. The authors should provide a more in-depth analysis of the significance of the minimum, maximum, mean, and standard deviation values of the dependent and independent variables in the model.
- Specify the Multivariate regression methods more explicitly in Table 5.

**Author response:** Based on the reviewer's suggestions, in the research findings section, we provided clear conclusions about the assumptions and discussed their consistency with previous research.

Mark the significance below each table. Table 3 provides a more detailed description. In the model section of the article, it was stated that a fixed effects model was used.

**Author action:** The following is a modified portion of the paper in response to the comments：

This confirms hypothesis H_1_, and this finding is consistent with the results of Long et al. [14] who verified the effect of state-owned equity on corporate EID, but the difference is that this study tests the state-owned equity involved in private firms, and it shows that mixed equity structure still promotes EID in private firms. Column (3) tests the moderating impact of media attention on state capital participation and EID; state and media had a 0.042 interaction term coefficient, which was significant at the 5% level, indicating support for hypothesis H_2_. This finding is consistent with the findings of previous studies [49, 52]. Column (4) tests the moderating effect of appointed directors, supervisors, and executives on state capital participation and environmental information disclosure, at the 5% level, the State and Government interaction term coefficient was significant at 0.034. The findings suggest that the appointment of directors, supervisors and executives can positively moderate the connection between the usage of state-owned capital and the disclosure of environmental information. This also provides empirical evidence of the involvement of state-owned capital in the governance of private enterprises.

Table 3 displays the descriptive statistics for the main variables. From Table 3, we know that the mean value of the EID index of private enterprises was 0.134, and 0.147 was the standard deviation. The maximum value was 0.655, which shows that private enterprises' overall EID level was low. The average number of state in private enterprises was 0.019, with a standard deviation of 0.041, showing that state ownership of capital in private companies is low and a maximum shareholding of 0.240, proving that in various companies, the proportion of state-owned participants' shares varies greatly.

**Reviewer#1, Concern # 6: ENGLISH LANGUAGE AND STYLE:**

It has some minor mistakes. The article could be proofread by a native speaker to enhance the quality of communication.

**Author response:** The article has been professionally polished.

**Reviewer#1, Concern # 7: OTHER SUGGESTIONS FOR THE AUTHOR(S):**

-	Keywords should be arranged in alphabetical order.

-	The list of references should adhere to the required citation style. For example:

**Author response:** The keywords have been arranged in alphabetical order. And the references of the article has been modified as required.

**Author action:** Environmental information disclosure, Mixed ownership reform, Private enterprises, Profitability, State-owned capital participation

References for example:

1. Zhang Z, Su Z, Wang K, et al. Corporate environmental information disclosure and stock price crash risk: Evidence from Chinese listed heavily polluting companies. Energy Economics. 2022; 112: 106116.
2. Ikram M, Qayyum A, Mehmood O, et al. Assessment of the effectiveness and the adaption of CSR management system in food industry: The case of the South Asian versus the Western food companies. SAGE Open. 2020; 10(1): 2158244019901250.
3. Zhu Q, Cordeiro J, Sarkis J. Institutional pressures, dynamic capabilities and environmental management systems: Investigating the ISO 9000–Environmental management system implementation linkage. Journal of environmental management. 2013; 114: 232-242.

.........

**Reviewer#2, Concern # 1:** Concerns about environmental sustainability and corporate transparency.

The paper's overall structure is sound, with logical transitions between sections. However, some sentences are quite lengthy and complex.

**Author response:** Firstly, thank you to the reviewers for their recognition of the topic of the article. Secondly, we have sorted out the entire article, streamlined and polished it, and highlighted the details in red throughout the article.

**Reviewer#2, Concern # 2:** It is quite hard for reader to understand about the calculation of EID (EID = 100* ( EIDI/39)). why using EIDI divide 39?

And this measurement you create or take advance from prior research? Please explain more and citation (if have).

**Author response:** We have provided a detailed explanation of the optimal score for environmental information disclosure. And the citation of relevant literature has been added.

**Author action:** Referring to the research of scholars such as Chen, we constructed an evaluation index for environmental information disclosure and obtained an optimal score of 29 points. The detailed scores for each part are shown in Table 1. The environmental information disclosure score of the sample is divided by the optimal score to characterize the level of environmental information disclosure.

Table 1. Corporate EID indicators.

| Level 1 Indicators | Secondary indicators | Numerical value | | |
| --- | --- | --- | --- | --- |
|  |  | Undisclosed | Qualitative Disclosure | Quantitative Disclosure |
| Environmental Liabilities | Discharge of wastewater | 0 | 1 | 2 |
|  | Emissions of SO_2_ | 0 | 1 | 2 |
|  | Emissions of CO_2_ | 0 | 1 | 2 |
|  | Emissions of smoke and dust | 0 | 1 | 2 |
|  | Generation of solid waste in industry | 0 | 1 | 2 |
| Environmental Governance and Performance | Techniques for lowering emissions of exhaust gas | 0 | 1 | 2 |
|  | Wastewater abatement treatment | 0 | 1 | 2 |
|  | Cleaner production implementation | 0 | 1 | 2 |
|  | Use and disposal of solid waste. | 0 | 1 | 2 |
| Disclosure Vehicle | List Companies' Annual Reports | Have for 1, not have for 0 | | |
|  | Social Responsibility Report |  |  |  |
| Environmental Costs and Inputs | Environmental expenses |  |  |  |
|  | Total environmental investment |  |  |  |
| Environmental Management | Training and education in environmental studies |  |  |  |
|  | The environmental incident response mechanism |  |  |  |
|  | Environmental concept |  |  |  |
|  | Environmental goals |  |  |  |
| Government Regulation and Institutional Certification | The implementation of the " triple concurrent" system |  |  |  |
|  | ISO environmental certification implementation |  |  |  |
|  | Environmental honors |  |  |  |

**Reviewer#2, Concern # 3:** In the part Explanatory variables- State-owned capital participation (State), you mentioned "We do this by consulting prior research" but no reference cited here. Please provide reference.

**Author response:** As suggested by the reviewers, in the part Explanatory variables, we cited relevant references.

**Reviewer#2, Concern # 4:** It will be better when you explain more about the Moderating variables- Media attention and the methodology applied (Python programming).

**Author response:** For the measurement of the moderating variable media attention, this paper refers to the practice of Chinese scholars, just using Python software for statistical work on the number of media reports, it may be that the expression is not standardized, resulting in your misunderstanding, please understand that this paper has been professionally embellished.
