# Supplementary material for: Environmental disclosure practices in mixed ownership models: A study of Chinese private enterprises
Source: PLoS One. 2023 Nov 30;18(11):e0295399. doi: 10.1371/journal.pone.0295399 (PMC10688842; doi:10.1371/journal.pone.0295399)
Supplement: Attachment — Submitted filename: Review Result_PONE-D-23-25299R1.docx [file pone.0295399.s003.docx]

**REVIEW RESULT**

**Manuscript_PONE-D-23-25299R1: Environmental Disclosure Practices in Mixed Ownership Models: A Study of Chinese Private Enterprises**

Reviewer: Dr. Le Quynh Lien

1. **OBJECTIVE:** The objective of the article is met with the paper’s scope.
2. **INTRODUTION:** Authors added how this study contributes to the existing literature and elaborate on the novel contribution of this study compared to previous research.

**3.	LITERATURE REVIEW: PRIOR STUDIES AND HYPOTHESES DEVELOPMENT**

-	COMPREHENSIVE PRIOR LITERATURE: There are appropriate and adequate references to related and previous research. Literature review and hypothesis development discussed in more detail, in my opinion.

-	HYPOTHESIS DEVELOPMENT: The hypotheses have been developed based on previous studies.

**4.	METHODOLOGY, RESEARCH DESIGN AND METHODS**

-	The research methodology in this study is sound.

-	Authors explained why they chose to calculate the maximum score of 37 points for companies to determine their EID and then divide the EID scores of sample firms by this maximum score to obtain the company's EID index.

-	They enhanced the persuasiveness of using lag variables when conducting Robustness tests.

**5. FINDINGS & DISCUSSION**

-	They provided clearer conclusions about the assumptions and discussed their consistency with previous research.

**6. ENGLISH LANGUAGE AND STYLE:**

Keywords arranged in alphabetical order. The references of the article have been modified as required.
