# Supplementary material for: Environmental disclosure practices in mixed ownership models: A study of Chinese private enterprises
Source: PLoS One. 2023 Nov 30;18(11):e0295399. doi: 10.1371/journal.pone.0295399 (PMC10688842; doi:10.1371/journal.pone.0295399)
Supplement: Attachment — Submitted filename: Response to Reviewers.docx [file pone.0295399.s004.docx]

Response to Reviewers

Dear editor and reviewer:

Hello everyone, thank you very much to the editors and reviewers for carefully reviewing this article and providing valuable suggestions for revisions. I have made careful revisions and supplements according to the reviewer's comments, and all modifications have been highlighted in red font in the revised manuscript.

I have made revisions and supplements to the attached modification comments item by item, as follows:

**Additional Editor Comments, Concern # 1:** Instrumental variable (IV) approach: The author uses "average value of state-owned equity share of private enterprises in the same industry in the same year" as the IV. At this current form, the proposed IV cannot pass the exclusion restrictions of IV/2SLS. I suggest the author exclude the current firm before calculating the cross-sectional industry average of state ownership. That way is a trick to unlink the current firm's relatedness to that IV, thus rendering it more exogenous. Also, the author must report the under-identification, weak-identification test statistics, and the Anderson-Rubin confidence interval for the IV/2SLS estimation in Table 6.

**Author response:** Based on the reviewer's suggestion, I excluded the current firm before calculating the cross sectional industry average of state ownership. Then recalculated and updated Table 6 and related content, reporting the under identification, weak identification test statistics, and the Anderson Rubin confidence interval for the IV/2SLS estimation.

**Author action:** The following is a modified portion of the paper in response to the comments：

The average value (excluding sample enterprises for calculation) of state-owned equity share of private enterprises in the same industry in the same year was used as an instrumental variable and regressed by two-stage least squares estimation (2SLS). The under-identification test shows that the Anderson LM statistic is 248.87, p < 0.01. The estimation results reject the original hypothesis, indicating that the instrumental variable is correlated with the explanatory variables.The weak instrumental test for the 2SLS shows that the Cragg-Donald Wald F statistic is 252.90, which is greater than the critical value of 10%. Therefore, it is not a weak instrumental variable. Meanwhile, Anderson-Rubin wald also rejected the original hypothesis at the 1% level, indicating a strong correlation between instrumental variables and endogenous variables.

**Table 6. Instrumental variable results.**

| Variable | First-stage | | Second-stage | |
| --- | --- | --- | --- | --- |
|  | (1) | (2) | |  |
|  | State | EID | |  |
| IVState | 0.810*** |  | |  |
|  | (15.90) |  | |  |
| State |  | 1.031*** | |  |
|  |  | (4.89) | |  |
| Constant | -0.114*** | -0.535*** | |  |
|  | (-11.69) | (-13.31) | |  |
| Controls | Yes | Yes | |  |
| Year FE | Yes | Yes | |  |
| Industry FE | Yes | Yes | |  |
| N | 14339 | 14339 | |  |
| R^2^ | 0.080 | 0.089 | |  |
| First stage F value | 62.16 |  | |  |
| Cragg-Donald Wald F | 252.90 |  | |  |

Note: *** p<0.01, ** p<0.05, * p<0.1.

**Additional Editor Comments, Concern # 2:** Propensity Score Matching (PSM) approach: Please report the matching diagnostics (balance diagnostics) for the pre-match and post-match samples. Also, what happened if the state ownership is very small, like less than 5%? It is not significant to be considered a major shareholder. How do you clarify this?

**Author response:** Based on the reviewer's suggestion, I reported the matching diagnostics (balance diagnostics) for the pre-match and post-match samples. At the same time, I have carefully considered the possibility that state-owned equity may be very small, which cannot reflect this issue. I believe this issue is indeed worth my deep consideration. Through a review of existing literature, most scholars measure state-owned capital participation based on the proportion of China's equity held by the top ten shareholders of private enterprises. I believe another reason may be the issue of data acquisition. Obtaining all shareholder information in a company may be difficult, and I hope to solve this problem in future research. At the same time, refer to the research of Scholar Zhang.

**Author action:** The following is a modified portion of the paper in response to the comments：

We refered to the study of Zhang et al. [45], and set the private firms with state-owned equity holdings of more than 5% among the top 10 shareholders as the treatment group and value = 1. Otherwise, it is 0, which is regarded as the reference group. We used corporate size (Size), corporate age (Age), market competition (Com), cash flow (Cash), gearing ratio (Lev), return on total assets (Roa), percentage of independent directors (Indep), and other variables that may affect state capital participation in private firms. The results in Table 7 show that after the spread of all variables was less than 5% on the standard deviation scale, the balancing hypothesis was confirmed, even if there were substantial differences between the control and experimental groups before the matching process. This finding suggests that after propensity score matching, there was less gap between private enterprises that use state-owned capital and those that do not. Matching the regression results confirms the primary research findings (As shown in Table 8).

**Table 7. Estimation results and balance test of PSM**

|  | UnMatched | Mean | |  |  | t-tests | |
| --- | --- | --- | --- | --- | --- | --- | --- |
| Variable | Matched | Treated | Control | %bias | %reduct | t value | p > \|t\| |
| Size | U | 22.029 | 21.736 | 25.2 |  | 10.10 | 0.000 |
|  | M | 22.033 | 22.011 | 1.9 | 92.3 | 0.53 | 0.597 |
| Age | U | 18.726 | 16.303 | 40.8 |  | 15.79 | 0.000 |
|  | M | 18.719 | 18.715 | 0.1 | 99.8 | 0.02 | 0.984 |
| Com | U | 0.089 | 0.085 | 3.9 |  | 1.51 | 0.132 |
|  | M | 0.089 | 0.089 | 0.2 | 94.1 | 0.06 | 0.951 |
| Cash | U | 0.036 | 0.045 | -4.7 |  | -3.02 | 0.003 |
|  | M | 0.042 | 0.043 | -0.4 | 91.1 | -0.30 | 0.766 |
| Lev | U | 0.417 | 0.365 | 25.8 |  | 9.96 | 0.000 |
|  | M | 0.418 | 0.416 | 0.9 | 96.6 | 0.25 | 0.803 |
| Roa | U | 0.105 | 0.044 | 3.2 |  | 2.52 | 0.012 |
|  | M | 0.037 | 0.043 | -0.3 | 90.7 | -1.05 | 0.295 |
| Indep | U | 0.364 | 0.379 | -29.7 |  | -10.46 | 0.000 |
|  | M | 0.364 | 0.364 | 1.5 | 94.8 | 0.48 | 0.628 |

Note: *** p<0.01, ** p<0.05, * p<0.1.

**Table 8. Results of the robustness test.**

| Variable | (1) | (2) |
| --- | --- | --- |
|  | EID | EID |
| State | 0.115*** |  |
|  | (3.31) |  |
| Statedummy |  | 0.004* |
|  |  | (1.90) |
| Constant | -0.917*** | -0.828*** |
|  | (-16.61) | (-25.87) |
| Controls | Yes | Yes |
| Year FE | Yes | Yes |
| Industry FE | Yes | Yes |
| *N* | 4240 | 14339 |
| adj. *R*^2^ | 0.261 | 0.254 |

Note: *** p<0.01, ** p<0.05, * p<0.1.

**Additional Editor Comments, Concern # 3:**Why using two different explanatory variables ("State" & "State_")? The results of the two regressions in this table therefore cannot be comparable.

**Author response:** This article also uses the method of replacing explanatory variables to test robustness, where State_ The explanatory variable used to represent substitution may have some issues with the wording in the text. Therefore, I changed to Statedummy to represent the replaced explanatory variable and modified some of the textual content to make the logic clearer.

**Author action:** Finally, in order to exclude the impact of indicator selection on the research results, we replaced the explanatory variable of state-owned capital participation variable. We used the presence or absence of state-owned participating shareholders (Statedummy) among private companies' top ten shareholders firms as a proxy for state-owned capital participation, which is 1 when state-owned participating shareholders are present and 0 otherwise.The regression analysis was then restarted after the main core variables have been replaced. The regression results are shown in Table 8, maintaining the paper's ultimate conclusion.

**Additional Editor Comments, Concern # 4:** In tables: please clearly indicate the fixed effects by replace "Industry" with "Industry fixed effect", similar to "Year" changing to "Year fixed effect".
**Author response:** According to the reviewer's suggestion, I have changed the “Industry” and “Year” in each table to “Industry FE” and “Year FE”.

**Reviewer#1,**

1. OBJECTIVE: The objective of the article is met with the paper’s scope.
2. INTRODUTION: Authors added how this study contributes to the existing literature and elaborate on the novel contribution of this study compared to previous research.

3.	LITERATURE REVIEW: PRIOR STUDIES AND HYPOTHESES DEVELOPMENT

-	COMPREHENSIVE PRIOR LITERATURE: There are appropriate and adequate references to related and previous research. Literature review and hypothesis development discussed in more detail, in my opinion.

-	HYPOTHESIS DEVELOPMENT: The hypotheses have been developed based on previous studies.

4.	METHODOLOGY, RESEARCH DESIGN AND METHODS

-	The research methodology in this study is sound.

-	Authors explained why they chose to calculate the maximum score of 37 points for companies to determine their EID and then divide the EID scores of sample firms by this maximum score to obtain the company's EID index.

-	They enhanced the persuasiveness of using lag variables when conducting Robustness tests.

5. FINDINGS & DISCUSSION

-	They provided clearer conclusions about the assumptions and discussed their consistency with previous research.

6. ENGLISH LANGUAGE AND STYLE:

Keywords arranged in alphabetical order. The references of the article have been modified as required.

**Author response:** Thank you to the reviewer for accepting my revised article.

**Reviewer#2,**

Thank you for addressing all my comments. Overall, your study after revised is sound. It provides significantly contributes to understanding how state-owned capital participation affects environmental information disclosure in private enterprises.

**Author response:** Thank you to the reviewer for accepting my revised article.
